# Targeting mutant p53: Evaluation of novel anti-p53^R175H^ monoclonal antibodies as diagnostic tools

**DOI:** 10.1038/s41598-024-83871-w

**Published:** 2025-01-06

**Authors:** Diana Spiegelberg, Le-Ann Hwang, Khian Hong Pua, Sashwini Chandra Kumar, Xin Yu Koh, Xiao Hui Koh, Ram Kumar Selvaraju, Kanaga Sabapathy, Marika Nestor, David Lane

**Affiliations:** 1 Department of Immunology, Genetics, Pathology, Uppsala University, Uppsala, Sweden; 2 Department of Surgical Sciences, Uppsala University, Uppsala, Sweden; 3 Divisions of Cellular & Molecular Research, National Cancer Centre Singapore, Singapore, 168583 Singapore; 4 School of Biological Sciences, Nanyang Technological University, Singapore, 637551 Singapore; 5 Institute of Molecular and Cellular Biology, ASTAR, Singapore, 138673 Singapore; 6 Preclinical PET-MRI Platform, Part of Department of Medicinal Chemistry, Uppsala University, Uppsala, Sweden; 7 Department of Microbiology, Tumor and Cell Biology, Science for Life Laboratory, Karolinska Institutet, Stockholm, Sweden

**Keywords:** Cancer diagnostics, Mutant p53, Intracellular targets, Molecular imaging, Cancer, Biomarkers, Molecular medicine, Outcomes research, Preclinical research, Translational research, Oncology, Cancer imaging

## Abstract

About 50% of all cancers carry a mutation in p53 that impairs its tumor suppressor function. The p53 missense mutation p53^R175H^ (p53^R172H^ in mice) is a hotspot mutation in various cancer types. Therefore, monoclonal antibodies selectively targeting clinically relevant mutations like p53^R175H^ could prove immensely value. We aimed to evaluate the in vitro and in vivo binding properties of two novel anti-p53^R175H^ monoclonal antibodies and to assess their performance as agents for molecular imaging. In vitro, ^125^I-4H5 and ^125^I-7B9 demonstrated long shelf life and antigen-specific binding. Our in vivo study design allowed head-to-head comparison of the antibodies in a double tumor model using repeated SPECT/CT imaging, followed by biodistribution and autoradiography. Both tracers performed similarly, with marginally faster blood clearance for ^125^I-7B9. Repeated molecular imaging demonstrated suitable imaging characteristics for both antibodies, with the best contrast images occurring at 48 h post-injection. Significantly higher uptake was detected in the mut-p53-expressing tumors, confirmed by ex vivo autoradiography. We conclude that molecular imaging with an anti-p53^R175H^ tracer could be a promising approach for cancer diagnostics and could be further applied for patient stratification and treatment response monitoring of mutant p53-targeted therapeutics.

**Supplementary Information:**

The online version contains supplementary material available at 10.1038/s41598-024-83871-w.

## Introduction

p53 is the most mutated gene across all cancer types, and p53 mutations are heavily involved in carcinogenesis and the response to therapy^1^. About 50% of all cancers carry a mutation in p53 that impairs its tumor suppressor function, making p53 a rare, almost universal, cancer marker in an otherwise very heterogeneous landscape of oncogenes and tumor suppressor genes. Among the p53 mutations, several hotspot mutations occur frequently^2^. These include R175(4.8%), G245(3.12%), R248 (6.79%), R249 (2.59%), R273 (6.55%) and R282 (2.59%)^2,3^. The location of these mutations within the DNA-binding domain suggests their importance in disrupting the normal function of the p53 protein. There are generally two types of p53 mutants based on their effects on the protein structure and function. The first type, known as contact mutants, includes mutations like R248W and R273H^4^. These mutations specifically affect the ability of the mutant p53 protein to bind to DNA. As a result, mutant p53 loses its ability to carry out its tumor-suppressor functions, which include regulating cell growth, inducing cell death, and preventing the formation of cancerous cells. The second type of p53 mutants are conformational mutants, such as R175H and G245S^4^. These mutations alter the three-dimensional structure of the DNA-binding domain, rendering it incapable of properly interacting with DNA. Overall, both conformational and contact mutants of p53 result in defective DNA binding and the loss of p53’s tumor-suppressor functions. This disruption in p53 function allows cancer cells to proliferate uncontrollably and evade normal cellular mechanisms that would normally prevent tumor formation.

Furthermore, p53 mutations are involved in about 70% of Li-Fraumeni syndrome (LFS) patients, an inherited genetic disorder characterized by early onset of various types of sarcomas and carcinomas^5^. The p53 missense mutation (p53^R175H^) is one of the most common hotspot mutations of p53, and is also encountered in the germline of LFS patients. Gaining a deeper understanding of the entire spectrum of p53 mutations and their role in various cancer types remains a promising but difficult prospect, and understanding the biology of mutant p53 is fundamental to unveiling its role in carcinogenesis and response to therapy. Such understanding can also open the possibility of using specific single-amino-acid p53 variants as biomarkers for disease diagnostics, treatment follow-up, and targets for novel therapeutic solutions. One limiting factor has been the capacity for raising antibodies specific enough for mutant p53, since antibodies against single-amino-acid changes may be highly cross-reactive with wildtype p53.

However, recent advances in monoclonal antibody development have led to the generation of novel antibodies specific to p53 missense mutations, engineered to possess high binding affinity and selectivity for specific mutant p53 proteins^6^. The high specificity of monoclonal antibodies (mAbs) allows for the targeted binding to specific mutant p53 proteins and can help distinguish cancer cells from normal cells. These monoclonal antibodies have the potential to be used as vector molecules for therapeutic purposes but also molecular imaging techniques. By conjugating these antibodies with specific imaging probes such as radionuclides, they can be used as molecular imaging agents for the detection and visualization of mutant p53 in vivo by positron emission tomography (PET) or single-photon emission computed tomography (SPECT). Mutant p53 has been shown to have a half-life of several hours, compared to wild-type p53 of approximately 20 min^7^. As a result, these mutant proteins can accumulate in large quantities specifically in tumor cells, making them an even more attractive target for molecular imaging^8^. Targeting intracellular proteins with antibodies does however pose unique challenges since antibodies typically have limited access to the intracellular environment. Nonetheless, intracellular proteins can act as targets for therapy and imaging using specific antibodies if the antigen becomes accessible through cell permeability events occurring in tumor tissues. Furthermore, recent advancements in antibody engineering and delivery methods have opened up new possibilities for intracellular protein targeting, including protein-transduction domain fusion and nanoparticle-driven delivery^9^.

Consequently, if successful, molecular imaging offers the advantage of non-invasively visualizing and quantifying mutant p53 in cancer patients, allowing for early detection and precise characterization of cancerous lesions, guiding treatment decisions, and monitoring treatment response.

The present study aimed to for the first time to radiolabel two new and highly selective mAbs raised against p53^R175H^ (p53^R172H^ in mice) and to evaluate the in vitro and in vivo binding properties. The overall goal was to demonstrate the potential of these antibodies for cancer diagnostics, patient stratification, and treatment response monitoring in cases involving mutant p53.

## Results

### Ab selection and in vitro characterization of the mAbs 4H5 and 7B9

**Fig. 1:**
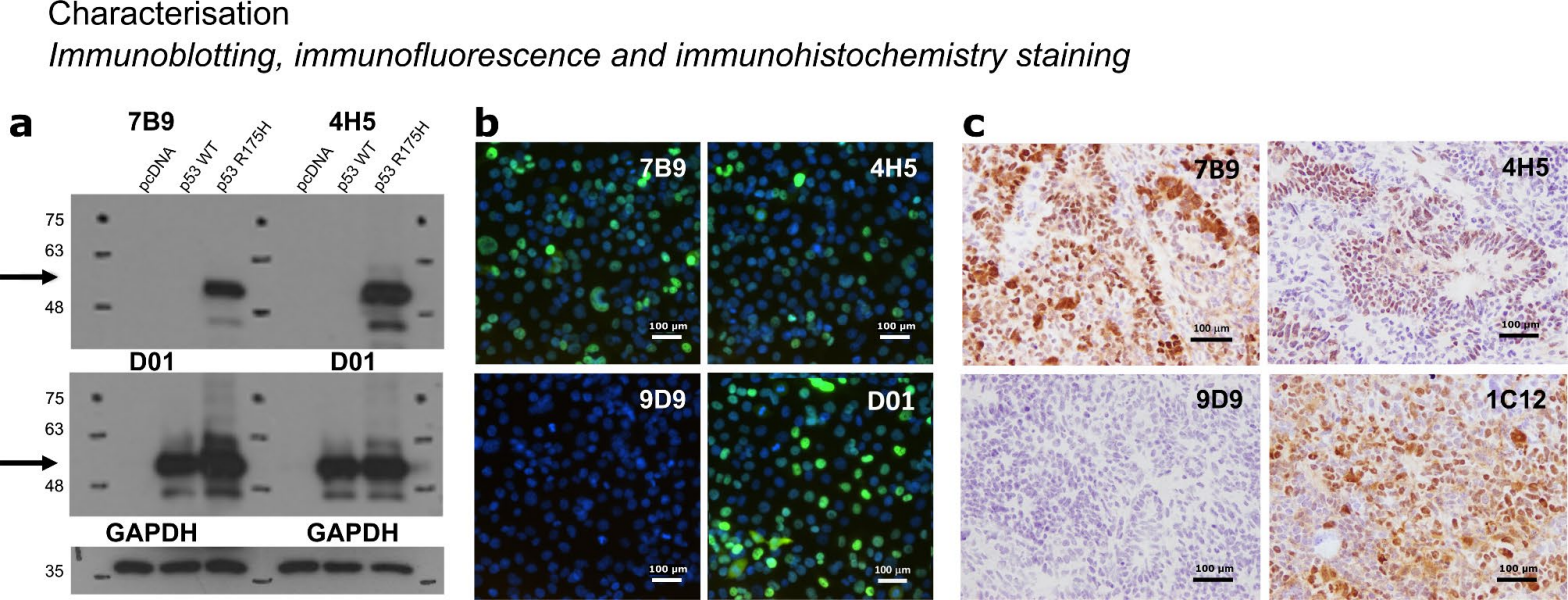
Characterization of anti-p53^R175H^ antibodies, by immunoblotting, immunofluorescent staining, and immunohistochemistry staining. (**a**) H1299 cells were transfected with indicated plasmids and equal amounts of lysates were used for immunoblotting with the 7B9, 4H5, DO1 (anti-human p53), and GAPDH antibodies. (**b**) Immunofluorescent detection of p53^R175H^ protein in p53^R175H^ transfected H1299 cells using 4H5, 7B9, 9D9 (anti-GST) and D01 mAbs. After p53^R175H^ transfection, cells were fixed in 4% paraformaldehyde and then permeabilized for mAb immunological fluorescent staining. (**c**) Detection of endogenous mouse p53^R172H^ protein in p53^R172H/R172H^ mouse tumor tissue section using Digoxigenin conjugated 4H5, 7B9, anti-GST control 9D9, or IC12 (against mouse and human p53) mAbs. Both 4H5 and 7B9 mAb specifically recognized and stained cell nuclei expressing endogenous mouse p53^R172H^ protein in tumor derived from p53^R172H/R172H^ mutant mice. Representative images are shown.

Initial antibody generation and characterization of the two R175H-specific antibody clones (7B9 and 4H5) has been previously described^6^. In brief, immunization with a fusion protein bearing multiple repeats of the p53 sequence focused around the R175H mutation elicited a mutant-specific antibody response, resulting in the generation of several hybridoma clones. The monoclonal antibodies (mAbs) only recognized the R175H mutant protein and not the wild-type p53 or other mutant p53 proteins with different amino acid substitutions. Immunoblotting using the 4H5, 7B9 antibody confirmed the specificity for the R175H mutant without any cross-reactivity to wild-type p53 (Fig. 1a, Supplementary Fig. 2a). Similar results were obtained by immunofluorescence staining using both the 7B9 and 4H5 clones (Fig. 1b). A quantitative comparison of the antibodies is provided in Supplementary Fig. 2b, showing the percentage of positively stained cells. Although no significant difference was detected between the performance of 4H5 and 7B9, the D01 antibody demonstrated higher staining levels. This discrepancy is likely due to differences in affinity of the mAbs or variability in transfection efficiency, as the p53-null cells were transfected with the p53-R175H mutant, potentially leading to inconsistent mutant p53 expression.

Immunohistochemistry results further confirmed the specificity of both 4H5 and 7B9 clones, upon conjugation with DIG, which eliminates non-specific-binding caused by secondary anti-mouse Ig, on mouse p53^R172H^ tumors (Fig. 1c). Thus, both the 7B9 and 4H5 clones were pursued for subsequent radio-labelling studies.

## In vitro characterization of radioiodinated 4H5 and 7B9

**Fig. 2:**
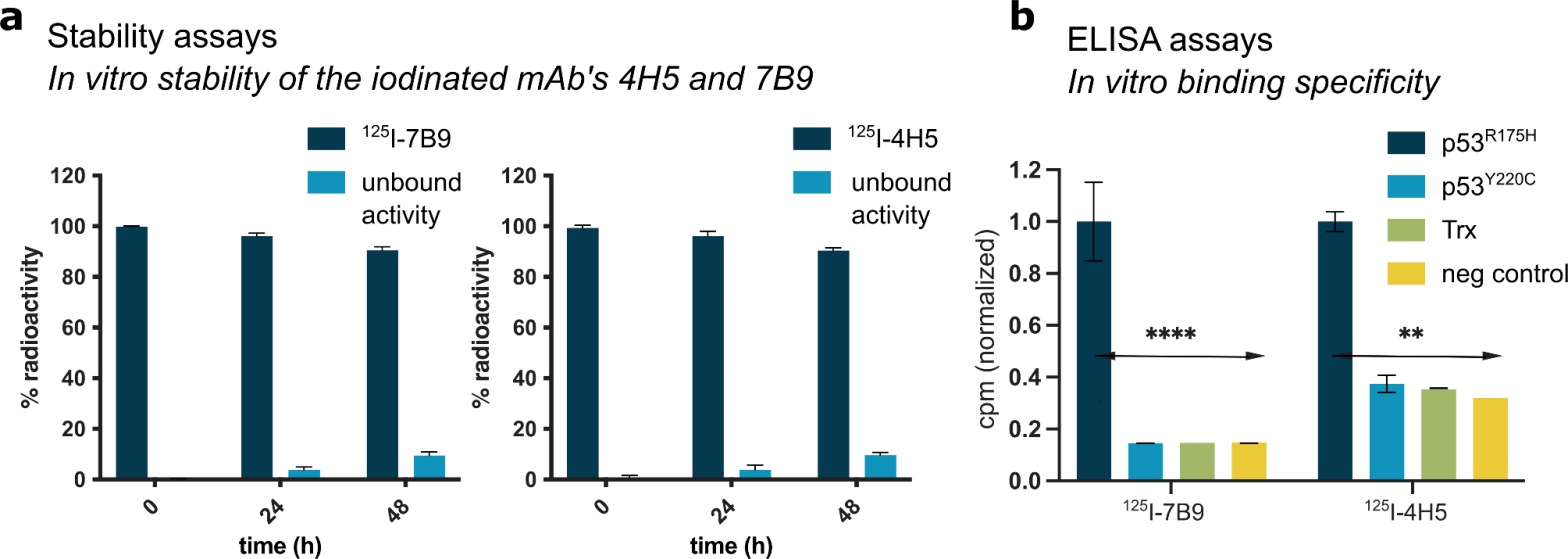
Stability and ELISA measurements of radioiodinated anti-p53R175H mAbs 4H5 and 7B9. (**a**) Stability measurement of ^125^I-4H5 and ^125^I-7B9 after 24 and 48 h post labelling (in PBS at room temperature). (**b**) In vitro binding properties of the radioiodinated anti-p53R175H mAbs 4H5 and 7B9 (10 nM) against mutated p53 forms and unrelated negative control proteins. ^125^I-4H5 and ^125^I-7B9 bound specifically to the mut p53^R175H^ protein. One-way ANOVA and Tukey’s multiple comparison posttest. **p ≤ 0.01 and **** for *p* ≤ 0.0001. Data are presented as the means ± standard deviation (SD), *n* = 3.

Radioiodination of the mAbs 4H5 and 7B9 with Iodine-125 (^125^I) using the Iodogen method resulted in high yield and purity (> 98%). In vitro characterizations of ^125^I-4H5 and ^125^I-7B9 demonstrated stability and long shelf life of the radio-conjugates (Fig. 2a). > 90% of both conjugates were still intact after 48 h at room temperature in PBS. ELISA assays with ^125^I-4H5 and ^125^I-7B9 against the p53^R175H^ protein, other different p53 mutations, as well as unrelated control proteins proved specificity for the R175H mutation for both antibodies as well as intact antigen-specific binding after the radiolabeling procedure (Fig. 2b).

## In vivo characterization of the mAbs 4H5 and 7B9

**Fig. 3:**
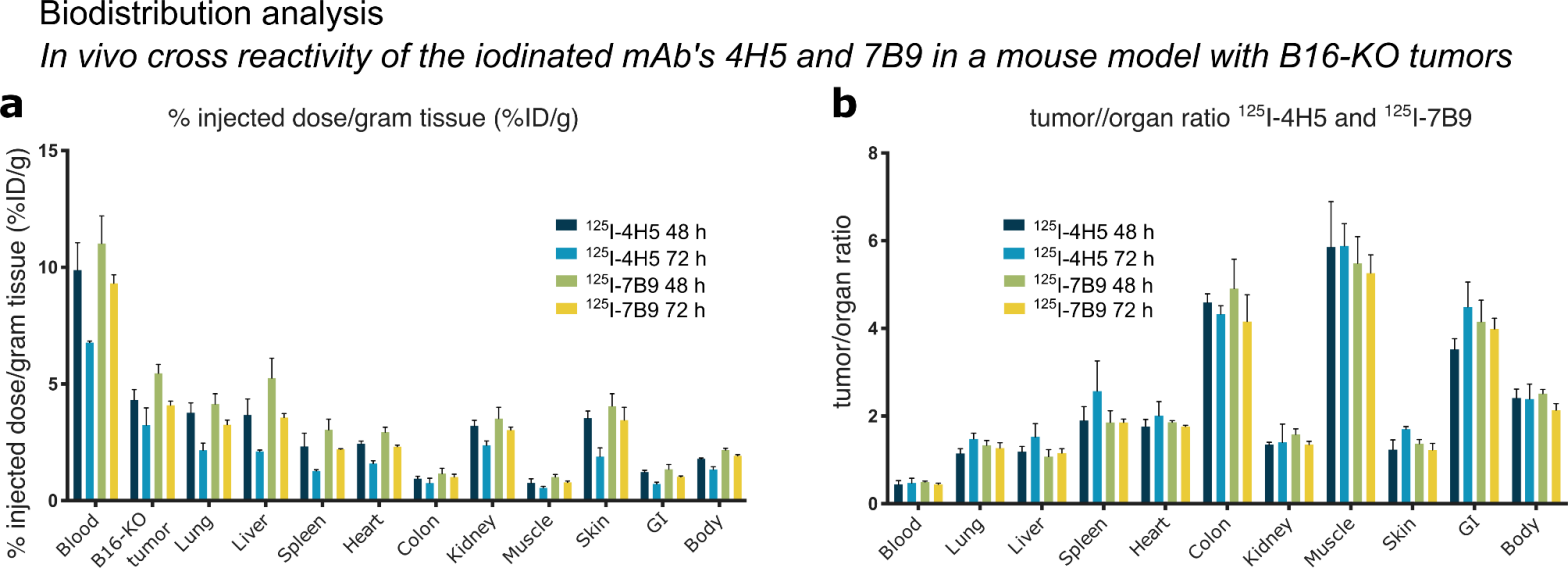
Biodistribution analysis. In vivo cross-reactivity analysis of the iodinated mAbs 4H5 and 7B9 in a mouse model with B16-KO tumors (8 mice/group). (**a**) % injected dose/gram tissue (%ID/g) and (**b**) tumor-to-organ ratios of ^125^I-4H5 and ^125^I-7B9 48 h and 72 h p.i. Data are presented as means ± standard deviation (SD).

In vivo, a cross-reactivity analysis of the radiolabeled mAb ^125^I-4H5 and ^125^I-7B9 (50 µg, 200 kBq) was first performed in a negative control mouse model that does not express any p53 protein with mouse melanoma B16 p53 knockout tumors (B16-KO) (Fig. 3). Biodistribution data collected 48 h and 72 h after tracer injection confirmed no non-specific accumulation of the tracers in any organ (Fig. 3a). ^125^I-4H5 and ^125^I-7B9 followed a similar distribution pattern. Furthermore, uptake of both radioconjugates in the p53 B16-KO tumors was comparable to tracer uptake in the other normal organs, except for blood (tumor-to-organ ratios of 0.44–0.49), probably due to the relatively long circulation time of full-size antibodies in the body (Fig. 3b).

**Fig. 4:**
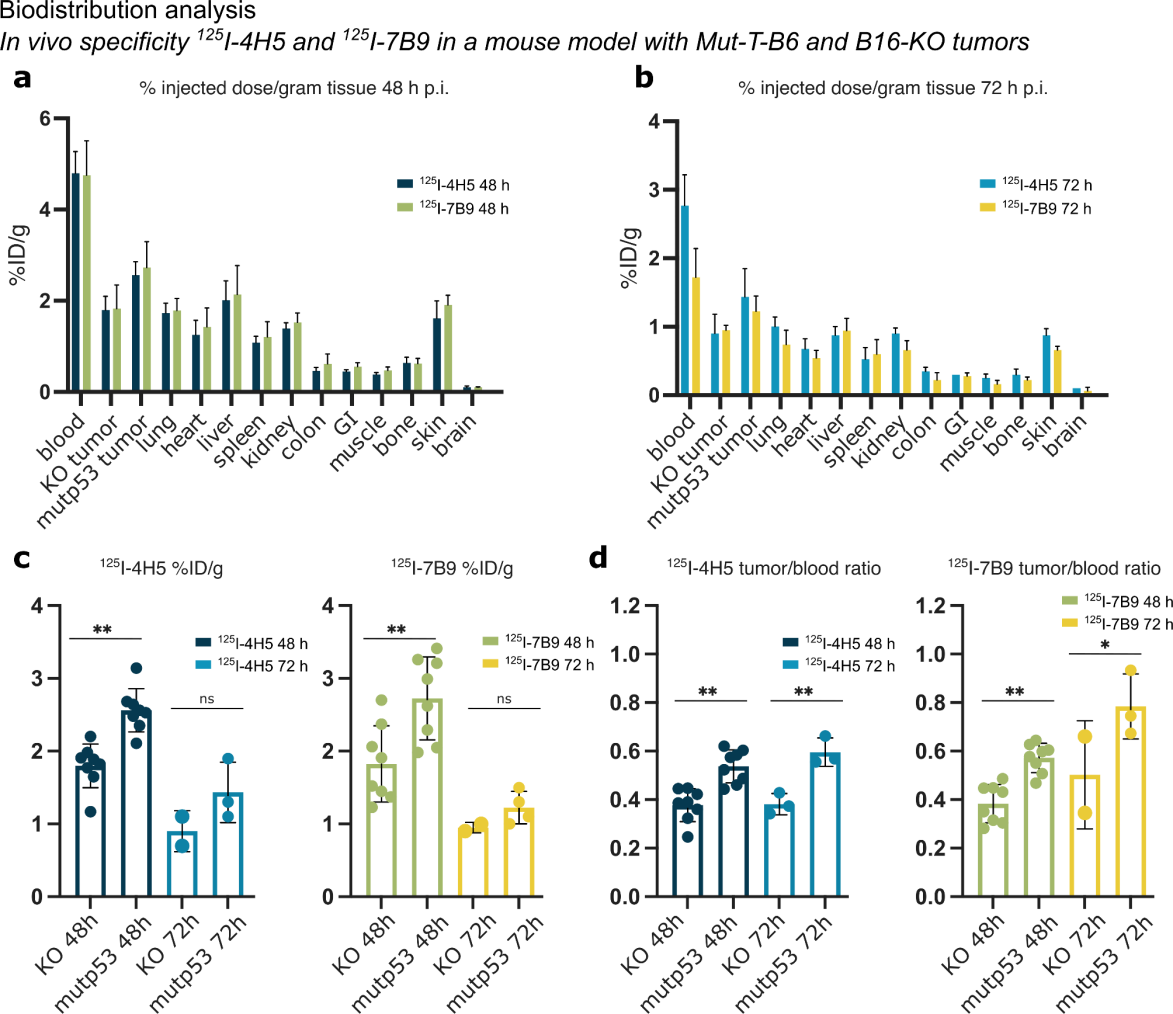
Biodistribution analysis. In vivo specificity of the iodinated mAbs 4H5 and 7B9 in a mouse model with Mut-T-B6 and B16-KO tumors. (**a**) % injected dose/gram tissue (%ID/g) 48 h (8 mice/group) and (**b**) 72 h (3 mice/group) p.i. of ^125^I-4H5 and ^125^I-7B9 for normal organs and tumors. (**c**) % injected dose/gram tissue for positive and negative tumors 48 h and 72 p.i. of ^125^I-4H5 and ^125^I-7B9. (**d**) Tumor/blood ratios for positive and negative tumors 48 h and 72 h p.i. of ^125^I-4H5 and ^125^I-7B9. One-way ANOVA and Tukey’s multiple comparison posttest. *p ≤ 0.05 and **p ≤ 0.01. Data are presented as means ± SD.

Next, the biodistribution of ^125^I-4H5 and ^125^I-7B9 (50 µg, 200 kBq) was evaluated in a dual tumor mouse model, with one mouse primary tumor (Mut-T-B6) expressing the mutp53 protein (p53^R172H/R172H^) on the right posterior flank and one p53 lacking mouse primary tumor B16-KO on the left (Fig. 4).

Similar to the previous cross-reactivity in vivo biodistribution experiment ^125^I-4H5 and ^125^I-7B9 showed comparable results (Fig. 4), high activity in blood and blood-rich organs with slightly faster blood clearance for ^125^I-7B9 compared to ^125^I-4H5 at the 72 h time point (from 4.8% ID/g at 48 h to 2.7% ID/g and 1.7% ID/g for ^125^I-4H5 and ^125^I-7B9, respectively). There was a significant difference in size between KO and Mut-T-B6 tumors (Supplementary Fig. 3a and b). However both ^125^I-4H5 and ^125^I-7B9 uptake (CPM and %ID/g) was significantly higher in the p53^R172H^ positive tumors compared to KO tumors 48 h p.i. This difference became less pronounced 72 h p.i. (Fig. 4a-c, Supplementary Fig. 3c and d). As a consequence of the remaining activity in the blood, tumor-to-blood ratios were < 1, however a significant difference between positive and negative tumor was observed for both investigated endpoints (Fig. 4d).

We further tested the CF750-conjugated 4H5 mAb in p53^R172H/R172H^ mutant B6 mice bearing spontaneous tumors. Mice were sacrificed 3 days post-injection for organ and tumor imaging using the IVIS Spectrum. As shown in Supplementary Fig. 4, we observed a strong signal in the tumors, along with some hotspots in normal tissues (carrying the R172H mutation), particularly in blood-rich organs like the kidneys and lungs. This could reflect either antibody uptake in these normal organs or elevated levels of circulating mAbs still present in the blood.

## Molecular imaging of ^125^I-4H5 and ^125^I-7B9 using repeated SPECT/CT scans

To study the in vivo imaging performance of ^125^I-4H5 and ^125^I-7B9 we used the same dual tumor model with p53 knockout and p53^R172H^ expressing mouse primary tumors, derived from p53 null and p53^R172H/R172H^ mice, as described in the biodistribution study.

Repeated molecular imaging of both tracers in 2 subjects scanned simultaneously with a SPECT/CT camera at 24 h, 48 h, and 6 d post-injection allowed head-to-head comparison of both iodinated antibodies in the same subjects over time. Both ^125^I-4H5 and ^125^I-7B9 demonstrated suitable imaging characteristics binding to the mutp53^R172H^ expressing tumors.

**Fig. 5:**
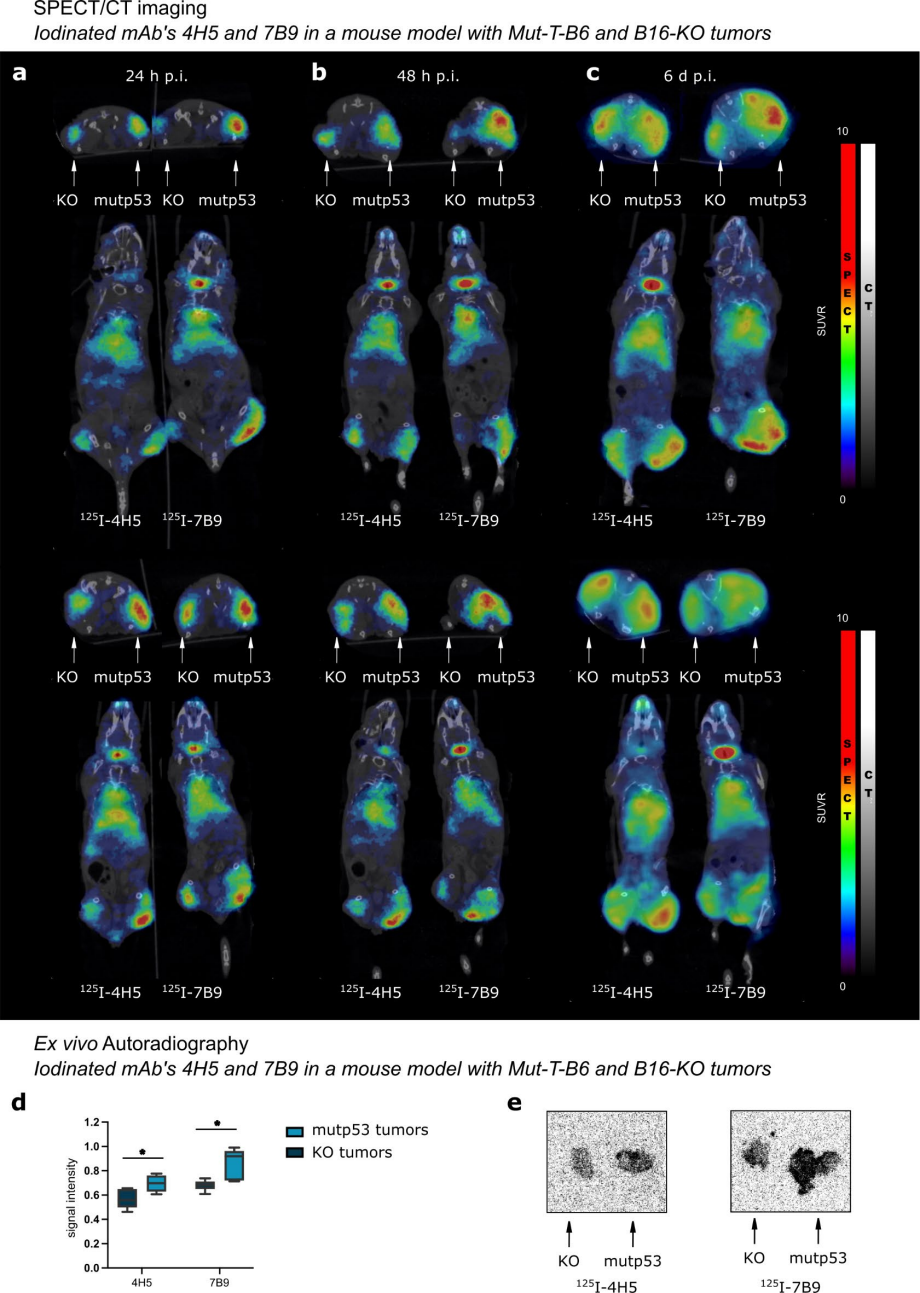
Repeated molecular imaging and analysis. Repeated SPECT/CT imaging of iodinated mAbs 4H5 and 7B9 in a mouse model with Mut-T-B6 and B16-KO tumors. 2 subjects were scanned simultaneously at (**a**) 24 h, (**b**) 48 h and (**c**) 6 d post-injection. The upper and lower rows represent the experimental replicates. SPECT images are presented in RGB and CT in gray color scale, respectively. (**d**) Ex vivo Autoradiography of iodinated mAbs 4H5 and 7B9 in Mut-T-B6 and B16-KO tumors 72 h p.i. (6–8 animals/group). One-way ANOVA and Tukey’s multiple comparison posttest. *p ≤ 0.05. Data are presented as means ± SD. (**e**) Representative images of ex vivo Autoradiography of iodinated mAbs 4H5 and 7B9 in Mut-T-B6 and B16-KO tumors 72 h p.i.

Figure 5a-c displays the SPECT/CT scans over time of four mice (2 for ^125^I-4H5 and 2 for ^125^I-7B9). Thyroid uptake was visible due to free ^125^I, while liver, lungs, and heart were visible most likely due to the slow blood clearance and slow secretion of the antibodies. The highest accumulation of ^125^I-4H5 and ^125^I-7B9 was detected in the mutp53 expressing tumors at all time points. Interestingly, some activity was already detected in the KO tumors at the time of 24-hour imaging, which remained at a similar level for 48-hour imaging, while activity in the positive tumors increased significantly from 24 to 48 h. This likely explains why the scans conducted at the 48-hour post-injection time poind yield images with the most pronounced contrast. Six days post injection, tracers remained detectable in both blood and tumors. Because imaging was conducted longitudinally, tumors had grown considerably larger by the 6-day time point compared to the earlier time points, influencing the biodistribution data where the injected activity is divided by tumor weight. SPECT images are presented in relative SUV scale (SUVR = 10) with respect to muscle.

## Autoradiography of ^125^I-4H5 and ^125^I-7B9 in KO and mutp53 tumors

Ex vivo autoradiography results confirmed the specific binding of both tracers to the p53^R172H^-positive tumors, significantly higher than the negative control tumors (Fig. 5d). However, uptake in the p53 null tumors (as also seen in the SPECT scans) was higher than expected. In both tumor models, the highest activity was measured at the rim of the tumor and in the narrow periphery of the blood vessels (Fig. 5e).

## Discussion and conclusion

Several hotspots in the p53 genetic sequence exist, where mutations are primarily encountered. Mutated p53 proteins encoded by these hotspot mutations almost always lose the functions of wild-type p53 and may instead guide functions associated with cancer development, such as promotion of proliferation, migration, initiation, invasion, angiogenesis, disruption of tissue architecture, and resistance to anticancer drugs^10^. The addiction of cancer cells to mutp53 makes it an attractive target for developing new diagnostic and therapeutic tools^11,12^. Furthermore, understanding the specific mutations and their functions can provide valuable insights for novel approaches to restore the tumor-suppressive properties of p53 in cancer treatment.

While techniques like next-generation sequencing (NGS) are crucial for identifying genetic mutations across the genome, they often require invasive biopsies and may not capture the full spectrum of genetic variation in all tumor lesions. Additionally, NGS provides a static snapshot of the tumor’s genetics and does not reflect real-time functional activity. In contrast, molecular imaging with specific tracers offers a non-invasive, real-time view of tumors, providing dynamic insights into the behavior of specific mutant proteins, such as R175H p53, within living organisms. This approach allows for the monitoring of changes in protein expression and tumor activity over time, providing functional data that complements the genetic information obtained from whole-genome sequencing.

In the present study, we evaluated the in vitro and in vivo binding properties of two monoclonal antibodies, 4H5 and 7B9, previously described in^6^, designed to target the p53R175H mutation, one of the most frequent p53 alterations in human cancers. The specificity of these antibodies was demonstrated through immunoblotting, immunofluorescence as well as ELISA techniques, showing recognition of the R175H mutant without cross-reactivity to wild-type or other mutant forms of p53. Radioiodinated ^125^I-4H5 and ^125^I-7B9 demonstrated long shelf life and antigen-specific binding. Our findings confirm the potential of these antibodies as agents for molecular imaging.

Several efforts have been undertaken, but mutant p53 is still difficult to drug due to a lack of thermostability and suitable binding pockets for small molecule drugs. However, generating mutation-specific reagents, like the monoclonal antibodies explored here, has significant potential for broad applications in biomedical research, supporting both basic studies and translational diagnostic and therapeutic efforts.

Most progress has been made targeting the Y220C mutation where both the pocket formed and reactive cysteine have been explored. For the Y220C mutation for example, an allele-specific reactivator drug rezatapopt (PC14586, mutant converted to WT activity) is currently in phase I and II clinical trials (NCT04585750)^13–15^. In preclinical studies, treatment with the small molecule rezatapopt induced complete regression of tumors in mice and potentiated checkpoint therapy. These experiments demonstrated that changing mutant p53 protein and restoring its wildtype function not only resulted in p53-mediated cell death but also improved the recognition and killing of the tumors by the immune system in small animal models. This included increased infiltration and activation of lymphocytes and other immune cells, similar to what was recently described for p53 activation in wildtype tumors using the stapled peptide Sulanemadlin^16^.

It is generally believed that antibodies typically require the target to be available extracellularly (e.g. on the outside of cell membranes or as a free molecule in the blood), with a concern that that antibodies targeting intracellular antigens may be hindered by the cell membrane. However, recent studies indicate that the integrity of cell membranes may be compromised within the tumor environment, thereby facilitating antibody access. For example, Dadachova et al. showed that a monoclonal antibody against melanin can deliver effective therapeutic doses of 188-rhenium to mouse melanoma tumor cells^17^. While the antibody showed an apparent preference for tumor cell melanin presumably because of accessibility caused by tumor cell membrane damage it is a concern that the antibody is not intrinsically tumor-specific. Here, we overcome this difficulty by using antibodies that are specific to tumor mutations in p53.

Complicating antibody recognition, specific p53 mutations may be masked intracellular by interactions with other proteins, such as p73^18,19^, potentially obscuring the R175H epitope. While the exact structure of the p53-p73 complex is unknown, studies suggest that p73 binds at the tetramerization domain (OD), distant from the DNA-binding domain (DBD) where R175H is located, indicating that the R175H epitope remains accessible^18^. Additionally, phosphorylated wild-type p53 is thought to bind p73 without blocking the DBD, suggesting that mutant p53 may behave similarly. Thus, antibodies like 4H5 and 7B9 should still effectively recognize and bind to mutant p53.

Another major challenge of antibody-based imaging techniques is the conjugation of the mAb with the radionuclide. Numerous direct and indirect labeling techniques can be used, however, only a fraction of these can be used without compromising the functionality and orientation of the mAb. Direct radioiodine labeling with Iodine-125, as performed in the current study, requires the presence of an aromatic moiety such as tyrosine or histidine^20^. Tyrosine is the primary site of iodine addition, but if the pH is above 8.5, the secondary site on the histidine ring is preferred. Since tyrosine is likely to be present in the binding region, it is important to evaluate the immunoreactivity and high molar activity of the binder after labeling and purification^20^. We were able to radio-iodinate 4H5 and 7B9 with a high yield and long shelf life. Moreover, the two radio-iodinated mAbs, ^125^I-4H5 and ^125^I-7B9, demonstrated selective in vitro binding properties after radiolabeling.

While the use of ^125^I as an imaging agent in preclinical studies has many advantages for preclinical work, particularly practical ones, such as availability and long half-life, there are also limitations. Due to the long half-time of 59.5 days and the relatively low gamma emission, ^125^I is rather impractical as an imaging agent in the clinical setting. However, ^125^I can be easily replaced by other halogen radioisotopes without changing the radiochemical labeling method, including ^124^I, which is suitable for PET imaging, and ^123^I, which is suitable for human SPECT imaging.

In this study, we could exclude in two separate small animal models any off-target accumulation in normal organs for both tracers. We observed a long circulation time in blood, which is often preferred in a therapeutic setting but is not optimal for diagnostic imaging.

Nevertheless, a significantly higher uptake of both ^125^I-4H5 and ^125^I-7B9 was observed in the p53^R172H^ expressing tumors at the tested time points.

In the in vivo study, a significant size difference between p53 KO and mutant p53 tumors was observed at 6 days post-injection, which complicates direct comparisons between the two models. To gain more insight into the specific impact of different p53 mutations on tumor behavior, further studies using tumors from the same cancer type with more similar growth rates would be beneficial. This could include tumors with other p53 hotspot mutations, such as R273H or G245S, to better understand how these mutations influence tumor growth and tracer uptake. Additionally, including immunocompetent mice in future studies would provide a more comprehensive evaluation of the immune response in these models. Together, these approaches would allow for more precise comparisons and help clarify the contribution of p53 mutation status to therapeutic outcomes and imaging biomarker efficacy.

Although the double tumor model used in this study is not ideal, using %ID/g accounts for size differences and shows higher tracer uptake in Mut-T-B6 tumors, not just due to their larger size. Importantly, the increased growth of the positive tumors does not lead to overestimation of uptake per gram, as smaller tumors usually show higher uptake. Larger tumors generally exhibit lower uptake per gram due to reduced antibody penetration^21^. Therefore, the significant differences observed in tracer uptake between p53 KO and mutant tumors are even more remarkable, as the larger tumor size of the mutant group would have been expected to mask, rather than exaggerate, the differences.

In addition, elevated activity was observed in the thyroid gland, suggesting that in vivo deiodination may occur. Therefore, blocking the thyroid gland with e.g. perchlorate before imaging would be beneficial^22^. It is also important to note that SPECT analysis alone cannot differentiate between various cell populations within a tumor. Previous research has identified mutant p53 secreted into the tumor microenvironment via extracellular vesicles suggesting that macrophages could uptake the R175H mutant from this environment^23^. However, our prior analyses of paraffin-embedded tumors stained with 4H5 and 7B9 did not show any cross-reactivity with wild-type p53, other mutp53 hotspot mutants, or immune cells, such as macrophages^6^.

Despite the high activity in the blood pool, we were able to visualize the p53^R172H^ mutation by repeated SPECT/CT imaging with the best contrast at 48 h. In further studies, imaging performance could be improved by developing smaller tracer variants. In contrast to whole-size mAbs, engineered affibody molecules and antibody fragments such as minibodies, diabodies, single chain variable region fragments (scFvs), and nanobodies are much smaller but retain the essential specificities and affinities. Advantages of smaller tracers include shorter blood circulation times (hours rather than weeks) improving the signal-to-noise ratio, deeper tissue penetration, and enabling same-day imaging^9,24^. In addition, the lack of the Fc region also lowers the nonspecific binding of the fragment and therefore may improve image quality. Imaging tracers smaller than 60 kDa are preferably excreted via the renal system and are not metabolized or retained by the liver. However, compared to full-size antibodies, smaller molecules may have poorer affinities and lower overall tumor uptake^24^.

Autoradiography of the negative and positive tumors revealed an accumulation of tracers in the narrow periphery of blood vessels and at the edge around the tumor, a phenomenon that also deserves further investigation in follow-up studies. While mAbs have the ability to bind specifically to tumor cells, their initial diffusion from the vasculature into tumor tissue is similar to that of non-targeting molecules and relies on the enhanced permeability and retention (EPR) effect, a unique property of tumors that affects diffusion of macromolecules through leaky blood vessels in the tumor area^25,26^. Due to the rapid growth of tumors, the surrounding blood vessels can have a defective architecture, which is exacerbated by the production of various permeability factors. Molecules larger than 40 kDa benefit from the EPR effect, promoting the accumulation in the tumor. However, the efficacy varies and is influenced by multiple factors including vascular permeability, endothelial receptors, vascular maturation, extracellular matrix, hypoxia, interstitial fluid pressure, and tumor cell density^26^. Strategies that promote EPR and release mAbs accumulated at the periphery of blood vessels could improve the access and availability of the mAb to the target. In agreement with this, we hypothesize that the uptake observed in the p53 negative tumors is due to the EPR effect may be reduced by using molecules smaller than 40 kDa. Here, for example, the use of Fab fragments could potentially achieve even better contrast between positive and negative tumors and could instead be better suited for diagnostic imaging.

While our study highlights the R175H mutation linked to LFS, we recognize that antibodies may not be suitable for treating LFS patients due to the presence of mutant p53 in healthy tissues. Our preliminary optical imaging results in R172H mutant mice using the CF750-conjugated 4H5 mAb showed greater uptake in spontaneous tumors, but also some uptake was also observed in normal organs (carrying the R172H mutation). Nonetheless, mutation specific antibodies could still be valuable for ex vivo applications, such as IHC, to detect and assess mutant p53 in LFS biopsy samples, thereby aiding diagnosis and research into the molecular landscape of LFS tumors.

This proof-of-concept study shows encouraging initial results and we can conclude that antibody targeting of intracellular proteins holds great potential for precision medicine and therapeutics. We believe that molecular imaging with anti-p53^R175H^ tracer could be a promising approach for cancer diagnostics and could be further applied for patient stratification and treatment response monitoring of mut-p53-targeted therapeutics as companion diagnostics. Further research and development in antibody engineering, delivery systems, and optimization of intracellular protein targeting approaches will be key to unlocking the full potential of this emerging field.

## Methods

### p53^R175H^ targeting antibodies

Construction and production of the mAb clones 4H5 and 7B9 specific for the p53^R175H^ mutant protein was previously described in^6^. The hybridoma clones were adapted to serum-free media by serial passage. The supernatant media was harvested and purified by protein A chromatography. PAGE analysis of the purified antibody showed heavy and light chain bands and no contaminants. The heavy chain and light chain sequences of the antibodies have been cloned. The recombinant expression of these clones gave the same specificities as the parent hybridoma. Thus, the antibodies are sequence verified.

### Iodination of the mAbs 4H5 and 7B9

Reaction vessels were coated with 20 µg Iodogen (1,3,4,6-Tetrachloro-3α,6α-diphenylglycouril) dissolved in dichloromethane at 0.1 mg/mL. After evaporation, ^125^I and 4H5 or 7B9 (150 µg in PBS) were mixed and added in an equivalent volume as sodium phosphate buffer, pH 7.4. The mixture was incubated at room temperature for seven minutes and gently shaken every 30 s. The reaction mixture was then transferred to a new tube with 1 mL 0.05 M sodium phosphate and 5 M NaCl, pH 7.4. buffer. After 10 min, one mL 0.05 M sodium phosphate, 5% potassium iodide, and 0.5% BSA (w/v) buffer was added and the sample was mixed thoroughly. Labeled conjugates were separated from unreacted radionuclide and low molecular weight reaction components using NAP-5 columns pre-equilibrated with PBS. Labelling yield and stability of the mAbs were evaluated by instant thin-layer chromatography (ITLC, Biodex Medical Systems) with subsequent quantification in a phosphoimager (BAS-1800II, Fujifilm).

### ELISA assays

R175H-Trx, Y220C-Trx, and Trx proteins were coated on Nunc 96-stripwell plates at the concentration of 0.5 µg/mL. Plates were left overnight at 4 °C. The following day, plates were washed with 0.05% PBS-Tween 20 (PBS-T) and blocked with 5% FBS in PBS with 0.05% (w/v) Tween-20 (blocking buffer) for an hour at 37 °C and washed three times. ^125^I-4H5 and ^125^I-7B9 were diluted in PBS-T (1nM, 10nM, 100nM) and added to the wells for one hour at 37 °C. After three washes, individual wells were separated and the associated activity was counted in a gamma counter (1480 Wizard 3′, Wallace).

### Cell lines

The murine melanoma cell line (H-2b background) B16-F10 was purchased from ATCC. B16-KO was generated by CRISPR-targeting (Supplementary Fig. 1a). The sgRNA target sequences used for targeting Exon 1 of murine p53 are CACCGTGACACCCTGCTGGGAAGG and AAACCCTTCCCAGCAGGGTGTCAC was cloned into PX458 plasmid with spCas9. p53 knockout B16-F10 cell lines were generated by transfecting 1 µg of PX458_mmp53KO plasmid with ~ 1 × 10^6^ B16-F10 cells as per the manufacturer’s instruction (Lipofectamine 3000). After 48 h post-transfection, B16 cells were dissociated into single cells and FACS sorted (MoFlo XDP 4) into single cells, in wells containing DMEM media complete with 20% FCS. To screen the population for CRISPR knockout efficiency, genomic DNA from B16 cells was extracted using QuickExtract DNA solution (Epicenter) and primers flanking the targeted region were used in PCR to obtain amplicons that are subsequently subcloned into pCR-BluntII-TOPO vector (Life Technologies). The amplicons were checked by sequencing (using the primer pairs: Mmp53_Ex1_F: GTTCTGTAGCTTCAGTTCATTGG and Mmp53_Ex1_R: CCGAGAGGTCTCGTCACGCTC) to assess the efficiency of sgRNA targeting p53. Clonal cells possessing p53 knockout were checked by sequencing and confirmed via western blot and immunohistochemistry with the corresponding antibodies (Supplementary Fig. 1b).

The primary mouse p53^R172H/R172H^ cell line, referred to as Mut-T-B6, was established from a spontaneous primary sarcoma-like tumor in a p53^R172H/R172H^ mutant B6 mouse with H-2b background. These cells have been characterized, with data of the morphology and p53 expression status determined by Western blotting shown in Supplementary Fig. 1c and d.

All cell lines were grown in DMEM supplemented with 10% FBS and a mixture of streptomycin/penicillin, Mut-T-B6, and B16-KO with an additional 5% sodium pyruvate.

### Immunoblotting

Cell lysates were prepared by sonication in 0.1% Triton X PBS (PBST) with protease inhibitors. Protein concentration in cell lysates was determined using the QuickStart Bradford assay. 20g of cell lysates were mixed with NuPAGE LDS buffer, heated, and loaded onto 4–12% Mini-PROTEAN gels. Proteins were separated by electrophoresis and transferred to nitrocellulose membranes. Membranes were blocked in 5% milk in PBST. 7B9, 4H5, or other indicated antibodies were used as primary antibodies and detected with goat anti-mouse IgG (H + L) (1:3000, Jackson Laboratories). Chemiluminescence detection was performed using SuperSignal West Dura Substrate. Imaging was done with Licor Odyssey Fc and Image Studio software. For uncropped membranes see Supplementary Fig. 2.

### Immunofluorescence (IF) staining and immunohistochemistry (IHC) staining

Cells or frozen tumor tissue sections were fixed with 4% paraformaldehyde, permeabilized, and blocked non-specific binding with 5% BSA (for IF) or 5% normal goat serum (for IHC) in PBS. Apply the indicated primary antibody or Digoxigenin (DIG) conjugated antibody to the sections and incubate overnight. After rinsing samples to remove the unbound primary antibody, Alexa Fluor 488-conjugated -anti-mouse Ig (1:500, Invitrogen, California, USA) secondary antibodies were used for IF. After 1 h incubation, samples were rinsed and stained nuclei with DAPI before mounting the coverslip onto the slides using an anti-fade mounting medium. IF staining images were observed using an Olympus FV1000 upright confocal microscope. Quantification was performed using NIH ImageJ software, according to^27^. First, image contrast was enhanced (Image > Adjust > Brightness/Contrast) with images converted to 16-bit format. We applied thresholding (Image > Adjust > Threshold, “Dark Background”) to isolate cells, followed by watershed segmentation (Process > Binary > Watershed) to define cell edges. Cells were counted using Analyze > Analyze Particles. Total cell number (A) was obtained from DAPI-stained images of p53R175H-transfected H1299 cells, and mAb-positive cells (B) were quantified from corresponding FITC-stained images. The percentage of positive cells was calculated as: % Positive = (B / A) × 100.

For IHC, HRP conjugated -anti-DIG secondary antibody (1:50, Jackson ImmunoResearch Lab) was added. After 1 h incubation, samples were washed and DAB solution, with hydrogen peroxide, was added. HRP zcatalyzes DAB oxidation, forming a brown precipitate at the antigen sites for visualizing. IHC Images were captured with a Zeiss AxioImager upright microscope.

### Small animal studies

Female nu/nu Balb/c mice (*n* = 58 total, age = 6 weeks, obtained from Janvier-Labs) were housed under standard laboratory conditions and were given ad libitum access to a standard laboratory diet and water. All animal studies were carried out in accordance with relevant guidelines, regulations and ARRIVE guidelines. The methods and protocols utilized in the animal study complied with Swedish law and were approved by the Uppsala Committee of Animal Research Ethics, Uppsala, Sweden (permit C33/16). All animals were randomized to the treatment groups. At endpoint all animals were euthanized with a mixture of ketamine (250 mg/kg) and xylazine (25 mg/kg) solution followed by heart puncture.

Biodistribution in mice carrying p53 negative B16-KO tumors.

32 mice were used to analyze the in vivo cross-reactivity of 4H5 and 7B9 to xenografts carrying p53-negative B16-KO tumors. 1 × 10^6^ B16-KO cells were injected subcutaneously into the right posterior leg.

Biodistribution in mice carrying Mut-T-B6 and B16-KO tumors.

26 mice were used to analyze binding of 4H5 and 7B9 in mice carrying Mut-T-B6 and B16-KO tumors. 1 × 10^6^ Mut-T-B6 cells and 2 × 10^5^ B16-KO cells were injected subcutaneously in the lower left and right flank respectively.

50 µL ^125^I-4H5 or ^125^I-7B9 (both 200 kBq, 50 µg) was given intravenously. At 48 and 72 h post-injection (p.i.) blood, tumors heart, liver, kidneys, spleen, colon, stomach, GI, skin, bone, brain, and muscle were collected, weighed, and measured in a gamma well counter.

In vivo optical imaging of p53^R172H/R172H^ mutant B6 mice.

4H5 antibody was labeled with XenoLight CF750 using a Fluorescent Rapid Antibody Labeling Kit (Caliper Life Science, Hopkinton, MA, USA) according to the manufacturer’s instructions. The mutant p53^R172H/R172H^ mice were generated as previously described^28^. Two 6- to 8-month-old male mice bearing spontaneous tumors were used for tumor and organ imaging. The mice were intravenously injected with 50 ug XenoLight CF750- labelled 4H5 mAb in 100 µL PBS per mouse. Three days after the CF750-4H5 mAb injection, the mice were euthanatized by cervical dislocation which was done when the animal was fully anaesthetised with 2.5% Avertin. The injected mice were sacrificed 3 days after the injection, and the tumor, heart, lung, liver, spleen, kidney, intestine, colon and fat were scanned by Caliper IVIS image system (Perkin Elmer). The experiments were approved by the University Animal Care and Use Committee of A*STAR Biological Resource Center (Singapore), and were carried out in Biological Resource Center (Singapore) animal facility.

### SPECT/CT imaging

Mice carrying both Mut-T-B6 and B16-KO tumors (as described above) received an intravenous injection via the tail vein with 50 µl ^125^I-4H5 (7 MBq, 50 µg), or ^125^I-7B9 (200 kBq, 50 µg). At 24 h, 48 h, and 6 days h p.i. two animals (one for each tracer) were anaesthetized using isoflurane and imaged simultaneously with a static whole-body tomographical scan in the NanoScan SPECT/CT (Mediso Medical Imaging Systems Ltd., Hungary). First, a whole-body CT scan was acquired with the following parameters. Scan method: Semi circle FOV; projections 480; X-ray, 50 kVp and 600 µA; binning, 1:4; acquisition time, 2 min 47 s. SPECT scan was performed on the same scan range as CT, for 10 min with the following parameters. Frame time, 8 s; acquisition over 28,4 keV. SPECT raw data were reconstructed in Nucline software (3.04.015.000) using the TeraTomo 3D algorithm with 3 subsets, 48 iterations, and corrected for scattering and attenuation artifacts. The CT raw files were reconstructed using Filter Back Projection. SPECT and CT Dicom files were fused and analyzed using PMOD v 4.105 (PMOD Technologies Ltd., Switzerland).

### Autoradiography

72 h p.i., Mut-T-B6 and B16-KO tumors were collected from the animals included in the SPECT/CT imaging and embedded in an O.C.T medium (VWR, Belgium). and sectioned with a microtome (20-µm sections). The tumors were then sliced into 20-µm sections using a microtome, and the distribution of remaining radioactivity was captured with a phosphorimager (Fujifilm BAS-1800 II, Japan). ImageJ for Mac OSX version 1.48v (NIH, Bethesda, MD, USA) was employed to measure the activity distribution within the tumor Sect^29^. Activity was quantified as pixel intensity per tumor area in the phosphorimager output file, using an arbitrary scale and normalized to control.

### Statistical analysis

Data are presented as the means ± standard deviation (SD). Statistical analysis was conducted using GraphPad Prism 7 for Mac (GraphPad Software, CA, USA). Data was analyzed using an unpaired Students-Test for comparison of 2 groups, otherwise using One-way ANOVA and Tukey’s multiple comparison posttest. p-values of less than 0.05 were considered statistically significant. Asterisks indicate significance levels at * for *p* ≤ 0.05, ** for *p* ≤ 0.01, *** for *p* ≤ 0.001, and **** for *p* ≤ 0.0001.

## Electronic supplementary material

Below is the link to the electronic supplementary material.

Supplementary Material 1

## Data Availability

The datasets generated during and/or analysed during the current study are available from the corresponding author on reasonable request.
