# Supplementary material for: Targeting mutant p53: Evaluation of novel anti-p53R175H monoclonal antibodies as diagnostic tools
Source: Sci Rep. 2025 Jan 6;15:1000. doi: 10.1038/s41598-024-83871-w (PMC11704002; doi:10.1038/s41598-024-83871-w)
Supplement: Supplementary file 1 — Supplementary Material 1 [file 41598_2024_83871_MOESM1_ESM.pdf]

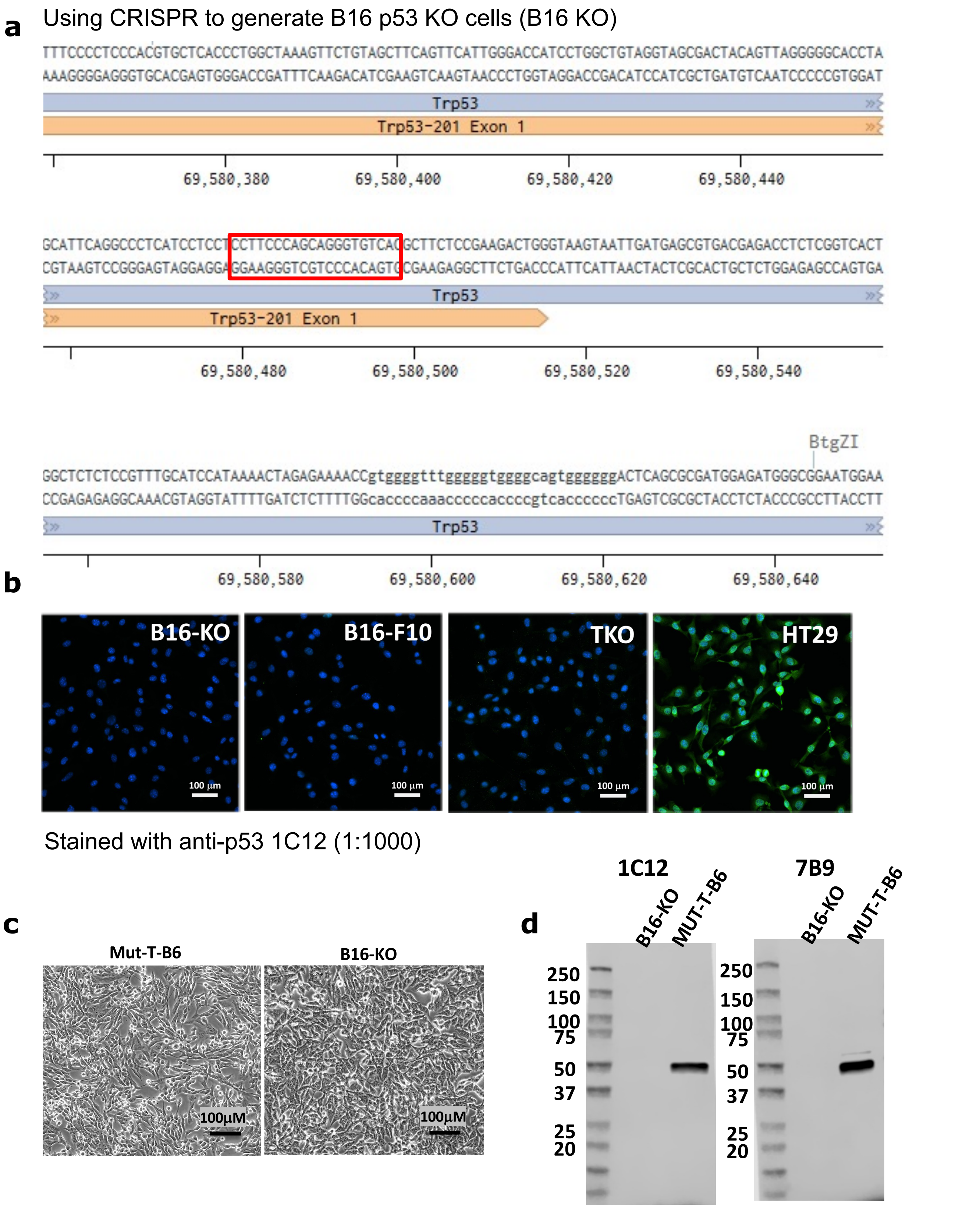

Supplementary Figure 1. p53 gene knockout by CRISPR/Cas9. (a) Sequence of exon 1 murine p53 being targeted by sgRNA (red box) in generating B16 p53 knockout cell line. (b) Immunofluorescence staining of p53 with IC12 anti-p53 antibody on the indicated cell lines. TKO: cell line lacking p53 as a negative control; HT29: p53-proficient human colorectal cell line as positive control. (c) Characterization of mouse p53R172H mutant (Mut-T-B6) and p53 KO cell line (B16-KO), cell morphology, bright field microscopy (10x). (d) Western blot analysis. Western blot analysis using 1C12 (anti-mouse and human p53) and 7B9 mAb on Mut-T-B6 and B16-KO cell lysate ran on 10% gel.

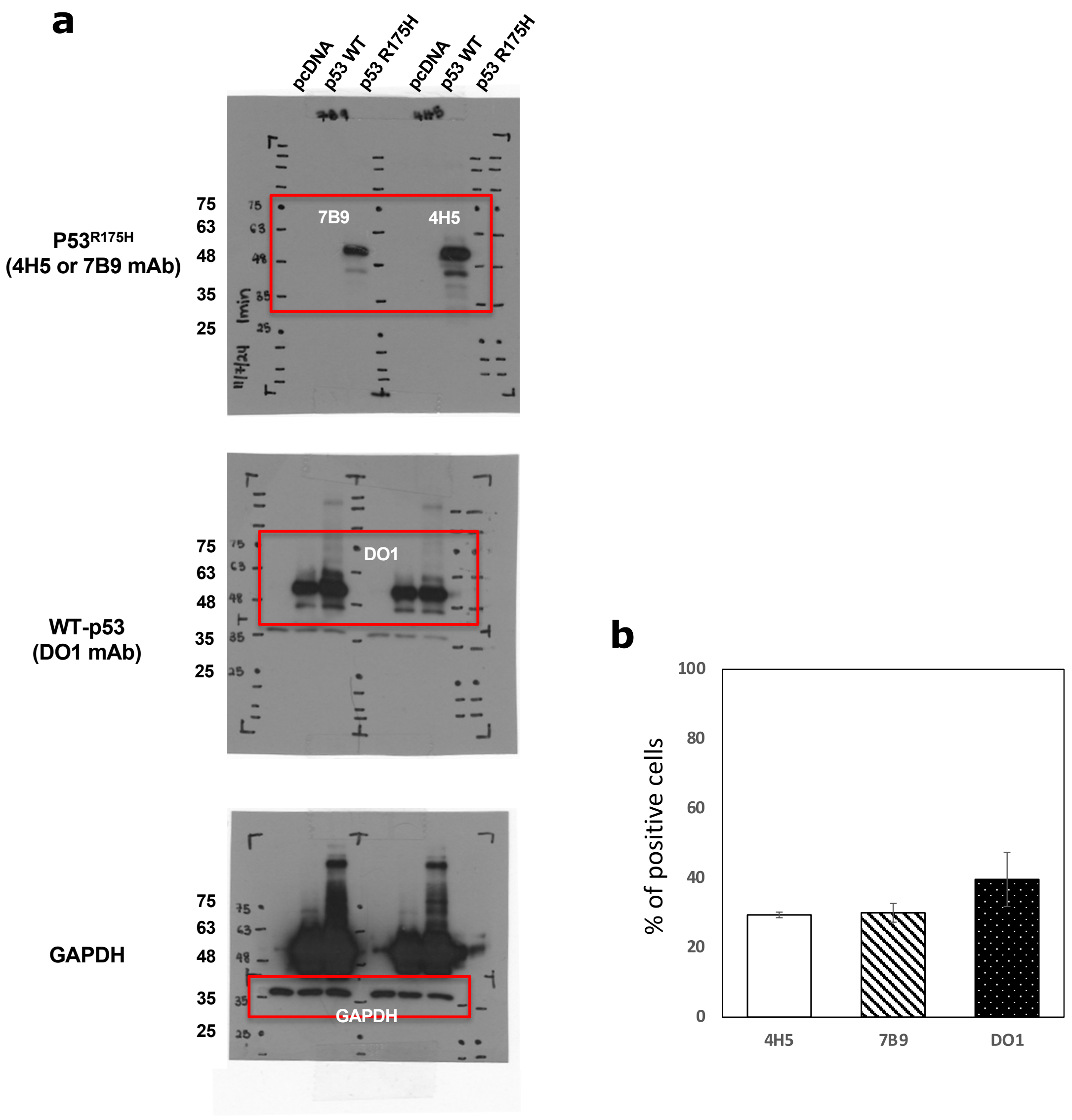

Supplementary Figure 2. Blot transparency and quantification of p53R175H transfected H1299 cells from Figure 1.
(a) Uncropped Western blot membranes. All primary data from Figure 1 are presented as uncropped images, with the relevant bands highlighted in red boxes. For the hybridization of p53 DO-1 and GAPDH antibodies, the membrane was cut in half, and the antibodies were probed separately but developed together. The p53 DO-1 (sc-126) and GAPDH (sc-47724) antibodies are well-established and have been cited in multiple publications.
(b) Quantification of p53R175H-transfected H1299 cells. Cells were stained with DO-1 (anti-wild type p53) and anti-p53R175H antibodies, 4H5 and 7B9. Quantification was performed using ImageJ software on confocal images, comparing cells stained with DO-1 to those stained with anti-p53R175H antibodies. The data, derived from Figure 1B and its replicates under the same staining conditions, showed no significant difference in the percentage of cells positive for anti-p53R175H versus anti-wild type p53 based on t-test analysis.

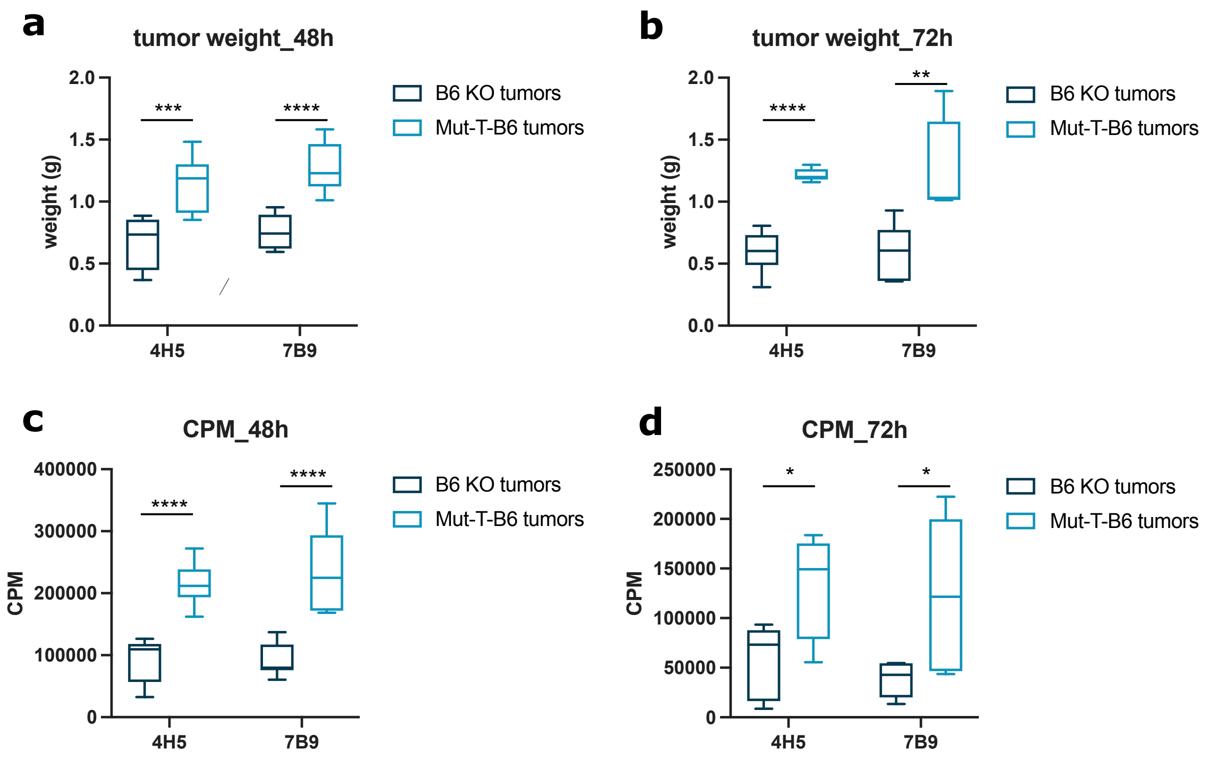

Supplementary Figure 3. Weight measurements and radioactivity counts per minute (CPM) of B6 KO and Mut-T-B6 tumors for biodistribution data shown in Figure 4.

Tumor weights for a) 48 h and b) 72 h post injection of 125I-4H9 and 125I-7B9. CPM for c) 48 h and d) 72 h post injection of 125I-4H9 and 125I-7B9. p-values of less than 0.05 were considered statistically significant. Asterisks indicate significance levels at * for $p \leq 0.05$, ** for $p \leq 0.01$, *** for $p \leq 0.001$, and **** for $p \leq 0.0001$..

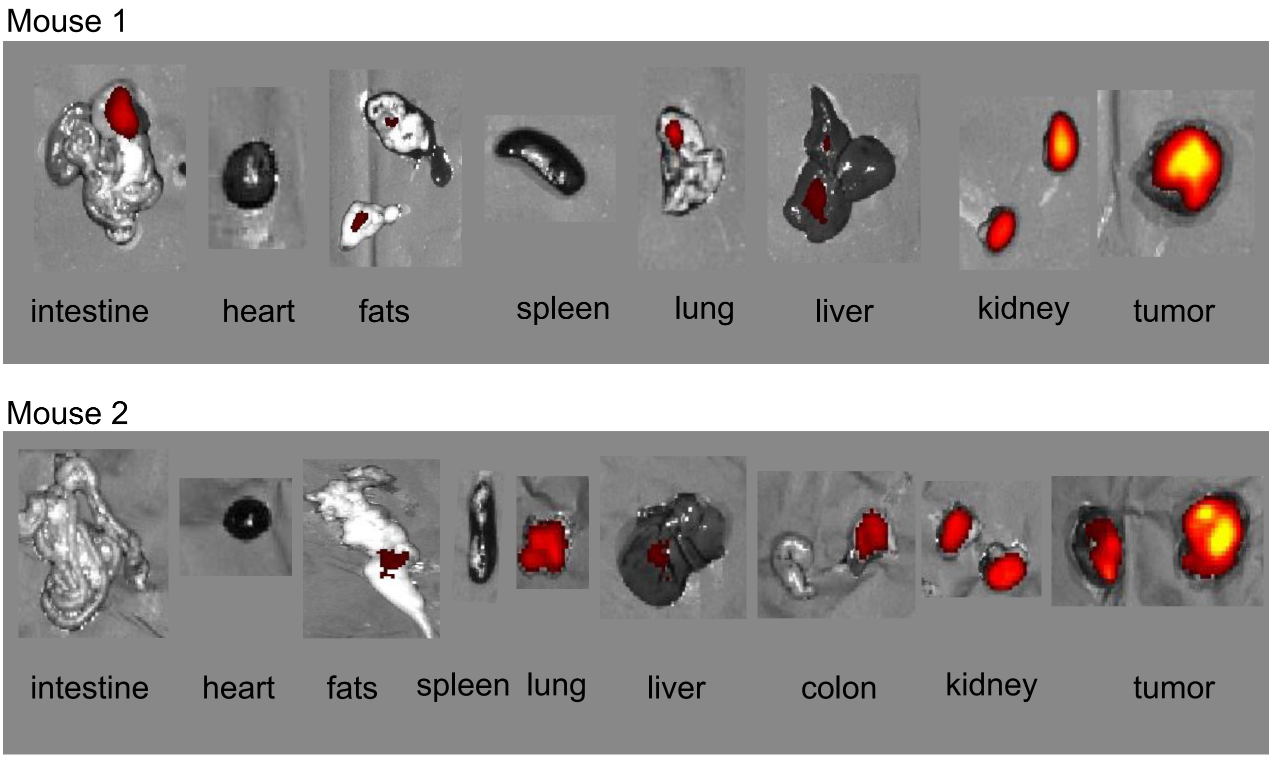

Supplementary Figure 4. CF750 conjugated 4H5 mAb was injected via tail vein in two tumor bearing gene modified mice, p53R172H/R172H mutant mice (mouse 1 and mouse 2) on day 0. Mice were sacrificed for organ and tumor imaging by IVIS spectrum on day 3.
